# Comprehensive linear and nonlinear analysis of the effects of spinning on dynamic balancing ability in Hungarian folk dancers

**DOI:** 10.1186/s13102-024-00850-4

**Published:** 2024-02-26

**Authors:** Zsófia Pálya, Rita M. Kiss

**Affiliations:** Department of Mechatronics, Optics and Engineering Informatics, Faculty of Mechanical Engineering, Budapest University of Technology and Economics, Muegyetem rkp.3., Budapest, H-1111 Hungary

**Keywords:** Hungarian folk dance, Dynamic balancing ability, Balance board, Nonlinear analysis, Fractal dimension, Sample entropy

## Abstract

**Purpose:**

In the case of Hungarian folk dancers, it is crucial to maintain correct posture and promptly respond to imbalances. However, traditional dances often lack specific training to develop these skills.

**Methods:**

In this present study, twelve dancers (8 male, 4 female, age: 21.7 ± 3.6 years) and ten non-dancers subjects forming a control group (6 male, 4 female, age: 21.6 ± 2.87 years) participated. During the measurements a 60-second long bipedal balancing test on the balance board was completed two times, and a spinning intervention was inserted in between the two sessions. The balance capabilities of the two groups were assessed through the characterization of motion on an unstable board, and the analysis of subject’s center of mass and head movements.

**Results:**

Dancers applied a more sophisticated and resource-intensive strategy to address the balancing task, yielding a better balancing performance in terms of balance board parameters. By preferring a solid stability in the medio-lateral direction, a greater fluctuation in the anterior-posterior direction can be observed (e.g., significantly lower *SampEn* values). The overall more successful performance is further evidenced by within-subject comparison since significant differences were observed mostly within the control group. Based on the results, the advanced balancing ability of the folk dancer group is more likely to be acquired through years of experience.

**Conclusion:**

The results indicate that additional specialized training could further enhance this ability, encouraging the reliance on poorly memorized corrective movements and reducing the risk of injury.

## Introduction

Hungarian folk dance is a collection of traditional dances originating in the Carpathian Basin. In the present day, regional folk dance styles have established dance sequences that require extensive practice to execute accurately, but they vary in their habitual and dynamic characteristics [1, 2]. Similarly to other traditional dance forms, dynamic styles involve elements like short jumps, leg swings, and leg slamming, which can increase the risk of injury [3], while other styles emphasize prolonged spins and rotations that demand strong balance skills [2, 4]. Engaging in spinning activities may have a negative impact on balance, potentially hindering the dancer’s performance and leading to injuries [5, 6]. Consequently, both postural control and fast postural responses are essential for dancers; thus, assessing athletes’ abilities and evaluating their balancing recovery efficacy is important [4, 6, 7].

Balancing ability of dancers is a widely examined field of scientific literature. The study of advanced postural stability and dynamic balancing skills has long been focused on ballet dancers [8–10]. However, contemporary research is expanding its scope to include other dance forms, acknowledging their significance in these skills. Bojanowska et al. studied Latin dancers’ static and dynamic balancing skills, and they found similar levels of dynamic balance in dancing people compared to non-dancers. Engaging in Latin dances may enhance static balance; however, this effect wasn’t extensively examined in the study and demanded further analysis [11]. Also studied Latin dancers by Liu et al., who found that dancers exhibited superior postural steadiness during the one-leg stance test, requiring less body adjustment for balance maintenance [12]. This finding is also significant clinically, as enhanced one-leg balance is crucial in preventing injurious falls, particularly in the elderly population. Limited literature exists on balance testing in folk dancers, primarily due to dance traditions’ diverse and nation-specific nature, leading to significant variations in dance styles. Quantitative analysis becomes challenging because of the absence of standardized rules for dance movements. According to Sevi et al., eight weeks of folk dance education can improve the balance. However, it remains unclear whether lifelong dance learning influences it [13]. Livska et al. examined the dynamic balancing stability of professional folk dancers compared to non-dancers. Folklore dancers exhibit poorer test results than the general population, potentially influenced by their specialized training in dance without focusing on improving general abilities [14].

Nonlinear analysis, utilizing parameters such as signal complexity and dimensionality, has allowed for the characterization of structural changes in postural control in conditions like low back pain, Parkinson’s disease, or patients with dysequilibrium symptoms or history [15–18], as well as during cognitive tasks or after sport-related concussions [19, 20]. However, traditional measures of postural stability are not sensitive enough to detect subtle changes, necessitating the use of dynamic approaches to extract physiologically meaningful information from stabilograms [21–23]. Recent studies have challenged the assumption that the variability of the center of pressure (CoP) during quiet standing is random, revealing a hidden structure and orderliness within apparently erratic CoP oscillations [20, 24, 25]. The complexity and regularity of CoP oscillations appear to break down with age and disease [26], indicating that relatively unconstrained and irregular CoP oscillations characterize optimal postural control. While the assessment of postural control through the variability of the CoP is common, recent approaches have sought to directly assess the complexity of postural control, which is closely linked to stability, adaptability, and coordination between system components. Following the successful implementation of nonlinear methods in the examination of static equilibrium, an increasing number of studies are being conducted to explore the fluctuations of the CoP in dynamic conditions [19, 21, 27–29].

The primary goal of this study is to conduct a comprehensive analysis of the balancing skill exhibited by Hungarian folk dancers by utilizing a multi-dimensional balancing device and spinning intervention. The present paper aims to uncover and substantiate the folk dancers’ presumed advanced level of dynamic balancing ability by applying a multilevel approach. Our primary purpose was to evaluate task success by applying metrics derived from the motion of the unstable balance board, thereby elucidating potential differences in stability between the anterior-posterior (AP) and medio-lateral (ML) directions across the two participant groups. Secondly, we seek to ascertain whether the observed device-induced variances are mirrored in the movement patterns of the participant’s motion. Alongside conventional parameters extracted from the "classical" CoP analysis (path, area of the confidence ellipse), nonlinear parameters such as sample entropy and fractal dimension are also involved, offering more insights into dynamic balancing performance. The anticipated outcomes are expected to reveal the advanced balancing capabilities of the dancers, showcasing their superiority compared to the control group. Furthermore, these results could be viewed not merely as definitive indicators but as catalysts for further refinement and development, aiming to propel balance skills for the dancers beyond current levels, transcending the memorized corrective movements achieved during practice sessions.

## Material and methods

### Subjects

Twelve dancers (8 male, 4 female, age: 21.7 ± 3.6 years; height: 175.4 ± 10.2 cm; weight: 72.5 ± 10 kg) and ten non-dancers subjects forming a control group (6 male, 4 female, age: 21.6 ± 2.87 years; height: 174.4 ± 7.82 cm; weight: 66.1 ± 11.56 kg) participated in the study. To participate in the study, dancers were required to have a minimum of eight years of dance practice, while the potential effects of training level, age, and gender were not accounted for. Dancers were sourced from university-related dance groups, with a usual background of learning Hungarian Folk dance since childhood and maintaining consistent participation in practices and rehearsals. In the selection process, it was assumed essential that years of experience were necessary to learn folk dance properly. Given that many dance clubs maintain training standards comparable to those of professional dancers, the criteria for selection emphasized active membership in a folk dance ensemble and a substantial number of years dedicated to Hungarian folk dance. Non-dancer participants were selected based on the criterion that they had yet to engage in high-level sports previously, which could markedly influence their balance ability nor were they currently involved in such sports. Subjects in both groups were excluded if they had any musculoskeletal injuries or disorders that could affect balance or locomotion. All participants have given their written consent to take part in the experiment after they were informed about all aspects of it. The protocol was approved by the Science and Research Ethics Committee of the University of Physical Education, Hungary (TE-KEB/17/2021).

### Dynamic balancing measurement

**Fig. 1:**
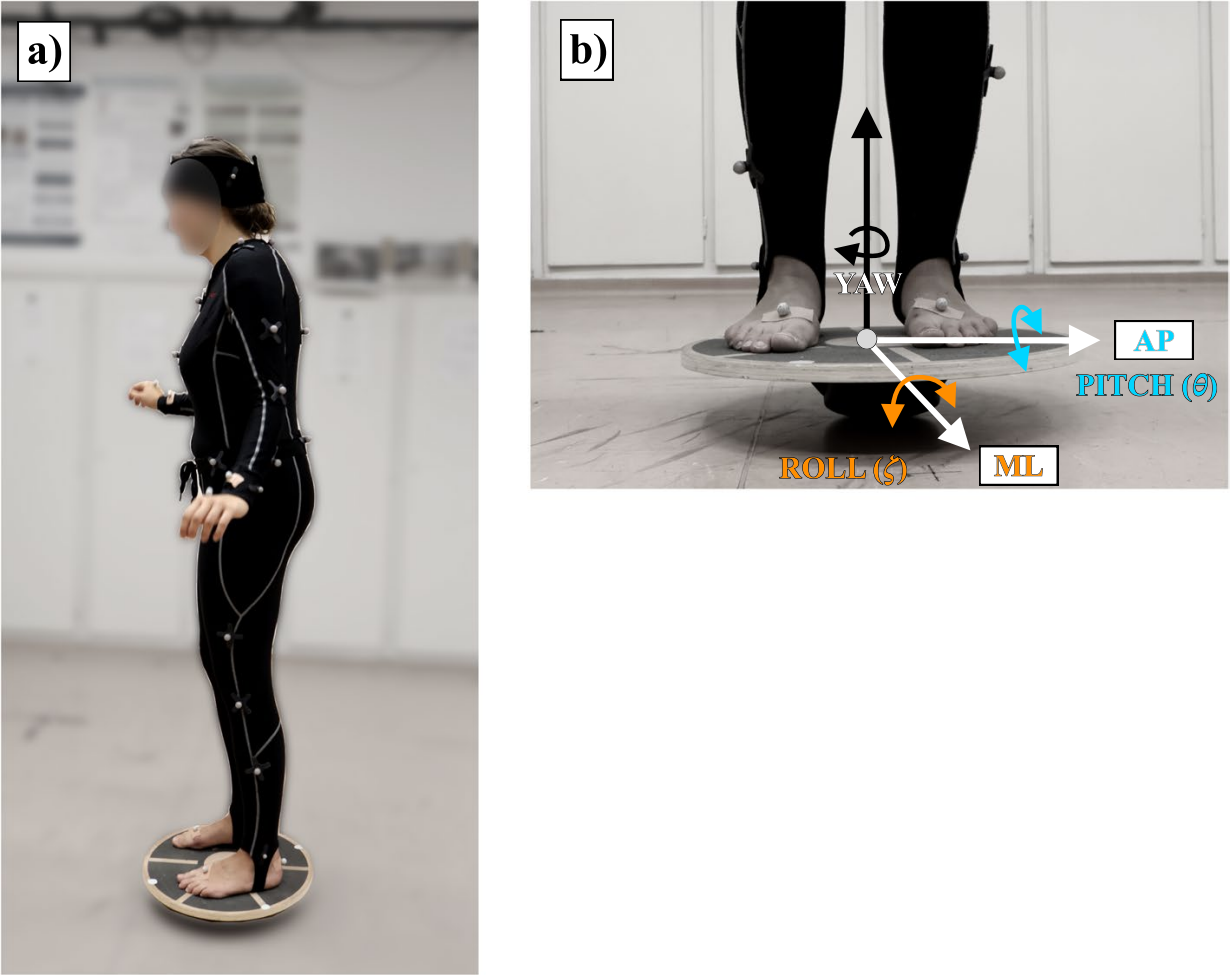
Measurement setup (**a**) and the definition of the roll, pitch and yaw angles (**b**)

A dynamic balancing task or test is an experimental procedure designed to evaluate an individual’s ability to maintain balance when one or several types of external perturbation or dynamic conditions are imposed upon it [30, 31]. During the measurements, participants were exposed to continuous dynamic excitation. A balancing test on the unstable board was performed to efficiently measure how effectively one could maintain balance on an unstable surface [32]. For the measurements, a multiaxial unstable balance board (DOMYOS, DECATHLON; Villeneuve-d’Ascq, France) with a diameter of 40 cm was applied. Participants were instructed to stand on the unstable board with eyes open while maintaining a steady posture as still as possible (Fig. 1). Moreover, an OptiTrack (NaturalPoint Inc.; Corvallis, Oregon, USA) three-dimensional optical motion capture system was used to record the motion of the balancing devices as well as the movements of the participants. The motion was recorded using 18 infrared Flex 13 (OptiTrack, NaturalPoint; Corvallis, Oregon, USA) cameras and the Motive v1.10.3 software. The software is responsible for the cameras’ coordinates operation and recording the markers’ three-dimensional position. This measuring system has an accuracy of sub-millimeter (overall Mean 3D Error: 0.550 mm) [33], and the measurements were carried out with a sampling frequency of 120 Hz. Spherical markers ($\varnothing$ 12.8 mm) covered with reflective coating were placed on the unstable balance board (five markers) to record its motion, whereas a full-body conventional marker-set was used consisting of 39 markers to capture the human motion [34]. The cameras cover a 4x2.5 m measuring area, with the unstable board in the middle.

### Experimental protocol

The measurement tasks were introduced to the participants, and some additional time was given to become acquainted with the measurement device and the spinner (EDEA Skates; Crocetta del Montello, Italy). The spinner (or spin-trainer, a foot-sized plate with a curved contact surface with the ground) is an ice sport-related device used to imitate the rotary motion, inducing some disorientation in participants. For the spinning intervention, a movement that is common in Hungarian folk dance and could be performed by both groups was sought. Spinning around the horizontal axis is a recurrent motif in folk dance, so quasi-steady rotation in the absence of a partner was feasible using the spinner. Subsequently, participants were asked to fill out the Waterloo Footedness Questionnaire to determine their dominant leg (20 participants were found strongly right-side dominant, and two participants were slightly more left-side dominant) [35]. The protocol started with the base measurements (*before* spinning), a 60-second long bipedal balancing test on the balance board. Upon that participants engaged in rotational activity (spinning intervention) by performing ten complete one-foot upright spins using the spinner device while standing on their dominant leg. The rotational fatigue session typically lasted between 30 and 60 seconds. Following the spinning activity, the 60-second long balance measurement was repeated to assess the post-spinning effects (*after* spinning). During the pilot phase of the study, the spin number was determined as the point where subjects had experienced a partial loss of orientation, yet were still capable of returning to the measuring device and completing the measurement. Note that to eliminate the cross-over effect, a 10-minute long break was inserted in between the base measurement and the spinning intervention.

### Data processing

The balance board has three degrees of freedom for rotation, which were consistently determined in relation to the subject’s position. Therefore, by utilizing anatomical markers on the participant, the balance board was rotated according to the anatomical planes in the AP (pitch), ML (roll), and frontal (yaw) directions (Fig. 1). Filtering positional marker data using a low-pass Butterworth filter (with a cut-off frequency of 5-6 Hz) has been a conventional approach in human gait analysis [36, 37]. Upon examination of our dataset, it was observed that smaller components contain valuable information beyond 6 Hz, and the signal is significantly distorted. Thus, our numerical data was processed using a fourth-order, low-pass Butterworth filter with a cut-off frequency of 15 Hz. To ensure consistency, the first and last five seconds of the 60-second trials were excluded from the analysis to eliminate any potential disturbances related to the balance board setup and landing. Moreover, due to the minimal friction between the ground and the bottom of the device, the rotational movement around the vertical axis (yaw) was found to be negligible during the measurements. Consequently, only the AP (pitch) and ML (roll) angles were considered for further analysis.

Two types of parameters were applied to characterize postural stability. The first type includes equilibrium parameters, which are directly associated with the stability of the balance board. These parameters are derived from the research conducted by Giboin et al., providing sensitive and reliable measures to describe the stability characteristics [38]. The equilibrium of the balance board (and therefore the subject) was defined by the parameters remaining inside the threshold zone. The threshold zone was calculated as the average value of rotation $\pm 10 \%$ of the range of the rotation similarly to [38], with our method being more permissive as it allows board stability not just in the horizontal plane. Practically, this translates to a change of approximately ± 2^∘^ from the average, considering a maximum deflection of around 20^∘^ in both directions. From the 50-second measurement interval, the range of angles (*RP* for pitch and *RR* for roll) and the percentage of stable regions (*SP* for pitch and *SR* for roll) were determined, from which the ratio of stable regions (*RSR*)

1 $$\begin{aligned} {RSR} = \frac{{SR}}{{SP}}, \end{aligned}$$

and the ratio between the two angle ranges (*RA*)

2 $$\begin{aligned} {RA} = \frac{{RR}}{{RP}} \end{aligned}$$

could be calculated. Furthermore, the settling times for the first occasion (*FSP* for pitch and *FSR* for roll) were also determined, which refers to the duration when the angular movement remains within the stable zone for at least two seconds.

Moreover, our objective was to assess the complexity of postural control more directly. Traditional methods for quantifying postural control primarily rely on assessing the CoP variability; however, in recent years, the use of wearable sensors and motion capture systems has gained popularity as these methods allow for the evaluation of posture stability in three dimensions [22, 39]. In the present study, 3D marker data was utilized as an alternative to the CoP, to estimate the subjects’ center of mass (CoM). CoM estimation was carried out by calculating the geometric center of four markers positioned on the pelvic girdle (*Left and Right Iliac Anterior Spine* and *Left and Right Iliac Posterior Spine*). In terms of linear parameters: CoM path (${P}_{CoM}$) and area of the 95$\%$ confidence ellipse (${A}_{95\%, CoM}$) were taking into consideration. According to Nagymate et al., the CoM path can be determined as at the length of total CoM trajectory during the measurement, whereas the confidence ellipse represents the smallest ellipse that covers 95$\%$ of the points [40]. The area of the ellipse was calculated using the eigenvalues of the covariance matrix. Investigating the movement pattern more specifically, we extended our analysis beyond the CoM to include head movement. Head motion was evaluated using four markers strategically positioned on the head, with two markers placed in front and two at the back. Similar to the characterization of CoM, the path length (${P}_{head}$) and the area of the 95$\%$ confidence ellipse (${A}_{95\%, head}$) was calculated for the head movement as well. This comprehensive examination gave us a detailed understanding of CoM and head movement patterns during the study. For the nonlinear analysis the calculated path lengths served as the basis. To gain a comprehensive understanding of the AP and ML movements, these components were examined individually during the analysis where two nonlinear indexes were chosen: sample entropy (*SampEn*) and fractal dimension (*FD*).

*SampEn*, is a valuable measure for evaluating the regularity of time-series data. It is calculated as the negative natural logarithm of the conditional probability that a dataset of length *N*, which has repeated itself within a tolerance *r* for *m* points, will also repeat itself for $m + 1$ points without allowing self-matches [41, 42]. This measure plays a significant role in analyzing postural sway regularity and is used to determine the stability and predictability of movements [22, 43]. *SampEn* in our case was determined using the method available on the PhysioNet tool [44]. According to the literature, the values $m=2$ and $r=0.2$ were selected for the sample size of $N=6000$ [22, 45].

**Fig. 2:**
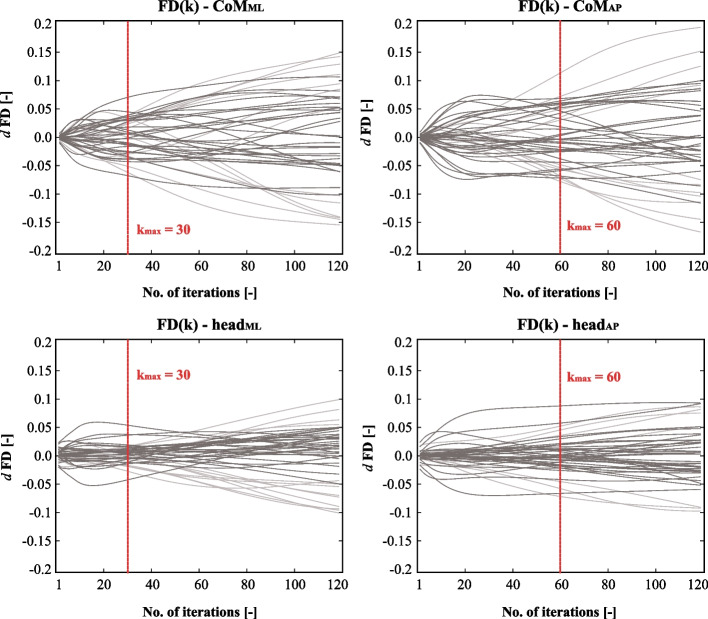
Change in the FD values of ${P}_{CoM}$ and ${P}_{head}$ both in AP and ML directions. The iteration was carried out for ${k}_{max} = 2,...,120$

*FD* serves as a measure for assessing the complexity of the CoP signal and describing its shape. Several algorithms are currently employed to calculate fractal dimension, including the Higuchi algorithm [46], Maragos and Sun algorithm [47], Katz algorithm [48], Petrosian algorithm [49] or, the box-counting method [50]. This measure reveals the degree of self-similarity and intricacy present in physiological signals. For calculating the *FD* of CoM and head path lengths Higuchi’s algorithm was used. Higuchi’s method [46] is a widely applied time-domain technique to determine fractal properties of complex non-periodic, nonstationary physical data. The Higuchi’s FD depends on the length of the time series and an internal tuning factor $k_{max}$. Higuchi’s original paper did not elaborate on the selection of the tuning factor, however, many examples can be found in previous articles [21, 51, 52]. Drawing from the work of Wanlissi et al., it is recommended to use a tuning factor within the range of $10^1$ and $10^2$ for a sample size of $N=6000$ [53]. To determine the suitable $k_{max}$ value, interpolation was conducted for each path trial for *k* ranging from 2 to 120. The difference between each interpolation point is depicted in Fig. 2. Based on the calculated results, $k_{max}=30$ for the AP direction and $k_{max}=60$ for the ML direction were selected. This decision was based on the observation that, for the vast majority of the time series, there were no significant changes in the FD values with respect to the subsequent iteration values.

### Statistical analyses

Before the statistical analysis, the calculated variables underwent normality tests. The normality of distributions was assessed with the widely-used Jarque-Bera test ($\alpha = 0.05$), since it is a quite robust test for smaller sample sizes and for slightly skewed distributions [54]. More than 80$\%$ of the balance board parameters successfully passed the normality test, however, only 15$\%$ of the linear and nonlinear CoM parameters passed the test. As a result, parametric tests assuming a normal distribution were applied for the balance board parameters. Whereas, the CoM parameters, which mostly failed the normality test, exhibited a non-normal distribution, and only non-parametric tests could be used. A between study design was adopted to compare the control and dancer groups both *before* and *after* the spinning intervention. For the balance board parameters, a two-sample T-test was applied, while for the CoM parameters, a Mann-Whitney U-test was performed. A within-subject study design was adopted to test the effect of the spinning intervention. Wilcoxon’s signed-rank test was used to compare *before* and *after* trials for the CoM parameters, and a one-sample paired T-test was performed on the balance board parameters. The effect size was determined to assess the outcomes of the statistical tests in-depth. The method of calculating and interpreting effect size differs between parametric and non-parametric tests, as outlined in Table 1. All the statistical tests were conducted at a significance level of $\alpha = 0.05$. All the statistical analysis were performed using the *Statistics Toolbox* of Matlab (version R2022b, The MathWorks Inc.; Natick, Massachusetts, USA).

**Table 1:** The predefined values were applied to interpret the effect size for each statistical test

	Parametric tests	Non-parametric tests	
	Cohen’s d (*d*)	Mann-Whitney U-test (*r*) [55]	Wilcoxon’s signed-rank test (|*r*|) [56]
Small	$0.20 - 0.49$	$\le 0.30$	$\sim 0.10$
Medium	$0.50 - 0.79$	$0.30 - 0.50$	$\sim 0.30$
Large	$\ge 0.80$	$\ge 0.50$	$\sim 0.50$

## Results

Based on the result of the between study design, there was no significant difference between the two groups in the balance board parameters (Table 2), as well as the CoM and head parameters (Table 3) before the spinning intervention (*before* condition). However, following the spinning, significant differences emerged in the ML direction stability ($p_{SR} = 0.0007$ and $p_{FSR} = 0.0368$) for the balance board parameters. Interestingly, the dancers exhibited an improved proportion of stable range (roll angle) and a decreased settling time for the the *FSR*, indicating enhanced stability despite the perturbation. In contrast, the control group showed a significant increase in the first set-up time, suggesting reduced stability. The effect sizes were large for both the *FSR* and *SR* parameters. Considering the linear parameters (Table 3), a notable difference between the groups was observed in head movement ($p_{Phead} = 0.0443$ and $p_{A95\%,head} = 0.0192$). The control group demonstrated a significant increase in the sway path and the area of the $95\%$ confidence ellipse. In contrast to the balance board parameters, for the nonlinear parameters, the significant differences between the groups were primarily associated with the AP direction of movement ($p_{SampEn,CoM,ML} = 0.0443$, $p_{SampEn,CoM,AP} = 0.0076$, $p_{SampEn,head,AP} = 0.0378$, $p_{FD,head,AP} = 0.0321$). The effect sizes were large for ${A}_{95\%, head}$ and ${SampEn}_{CoM,AP}$, and moderate for the other parameters. Entropy is a measure where smaller values indicate more regular motion, while higher values signify increased irregularity in the time series. Notably, in both AP and ML directions, the entropy values remained relatively close to the *before* values for the dancer group. However, for the control group, both the CoM and head motion showed a significant increase in entropy after the spinning intervention. This observation suggests that the control group’s motion became more irregular following the perturbation, in contrast to the dancer group, where motion regularity was largely maintained. In the case of Higuchi’s FD, a higher FD value typically indicates more self-similarity or smoother time-series data, while a lower FD value suggests less self-similarity or a more irregular and complex time series. Although the lower FD values for the control group are closer to perfect standing still condition, the higher value for the dancers implies a more organized time series. After the spinning intervention, the FD value for the control group either remained unchanged or decreased, whereas for the dancers, it increased in the AP direction.

**Table 2:** Balance board parameters of the study groups (control, dancer). Data are expressed as mean ± SD

	Control		Dancer	
	*before*	*after*	*before*	*after*
*RP* [^∘^]	21.51 ± 15.12	23.29 ± 12.88	26.37 ± 7.32	26.05 ± 9.48
*RR* [^∘^]	18.81 ± 14.37	23.97 ± 15.55	21.45 ± 5.02	29.01 ± 9.16 ^†^
*SP* [$\%$]	55.65 ± 21.93	49.49 ± 16.48	54.36 ± 12.14	60.63 ± 15.36
*SR* [$\%$]	57.36 ± 16.21	50.06 ± 15.21	59.25 ± 15.41	71.72 ± 10.21 ^∗,†^
*RSR* [-]	1.10 ± 0.25	1.05 ± 0.19	1.11 ± 0.26	1.23 ± 0.24
*RA* [-]	0.93 ± 0.34	0.94 ± 0.45	0.85 ± 0.21	1.19 ± 0.38 ^†^
*FSP* [s]	8.92 ± 10.38	8.85 ± 6.29	7.98 ± 5.48	9.55 ± 6.52
*FSR* [s]	3.29 ± 4.95	13.25 ± 8.25 ^†^	7.20 ± 5.96	6.82 ± 5.12 ^∗^

* *p* $<0.05$, group effect (two-sample t-test)

^†^ *p* $<0.05$, effect of the spinning (paired t-test)

The result of the within-subject design showed that the impact of the spinning activity on balance board parameters (Table 2) was more significant for the dancer group, particularly in the ML direction: roll range ($p_{RR} = 0.0387$), roll stable ratio ($p_{SR} = 0.0087$), and the ratio between the roll and pitch angle’s range ($p_{RA} = 0.0075$). All effect sizes were deemed large. Although the angular range of motion increased, which might suggest a potential decline in performance, a significant improvement was observed in the stable range of the roll angle, as well as the extent of the movement in this direction during the balancing activity. For the control group, a significant increase was observed in the first settling time of the roll angle ($p_{FSR} = 0.0030$). The statistical outcomes for linear and nonlinear CoM and head parameters (Table 3) exhibited distinctions from those obtained for the balance board parameters. Post-spinning differences were observed solely within the control group. In the linear case, the covered path lengths ($p_{PCoM} = 0.0195$ and $p_{Phead} = 0.0273$) increased significantly after the spinning intervention with large effect sizes. In the case of the nonlinear parameters, significant changes with large effect sizes were evident through increased entropy values ($p_{SampEn,CoM,ML} = 0.0195$, $p_{SampEn,CoM,AP} = 0.0488$ and $p_{SampEn,head,ML} = 0.0195$).

**Table 3:** Linear CoM and head parameters (path and area of the $95\%$ confidence ellipse) and the nonlinear CoM and head parameters (sample entropy and Higuchi’s fractal dimension) of the study groups. Data are expressed as median (interquartile range)

	Control		Dancer	
	*before*	*after*	*before*	*after*
${P}_{CoM}$ [cm]	95.04 (69.16)	165.90 (140.39) ^†^	82.86 (26.86)	92.84 (49.30)
${A}_{95\%,CoM}$ [cm^2^]	17.89 (66.83)	71.28 (199.60)	16.07 (11.22)	28.63 (102.87)
${P}_{head}$ [cm]	279.12 (186.83)	456.85 (390.08) ^†^	195.78 (92.20)	212.79 (79.98) ^∗^
${A}_{95\%,head}$ [cm^2^]	212.42 (504.58)	556.90 (990.77)	120.68 (131.21)	151.82 (134.70) ^∗^
${SampEn}_{CoM,ML}$ [-]	0.036 (0.007)	0.058 (0.053) ^†^	0.033 (0.012)	0.037 (0.019) ^∗^
${SampEn}_{CoM,AP}$ [-]	0.035 (0.024)	0.080 (0.049) ^†^	0.035 (0.013)	0.034 (0.017) ^∗^
${FD}_{CoM,ML}$ [-]	1.06 (0.02)	1.06 (0.03)	1.07 (0.04)	1.07 (0.03)
${FD}_{CoM,AP}$ [-]	1.08 (0.04)	1.07 (0.02)	1.09 (0.04)	1.10 (0.04) ^∗^
${SampEn}_{head,ML}$ [-]	0.087 (0.035)	0.164 (0.141) ^†^	0.073 (0.040)	0.071 (0.038)
${SampEn}_{head,AP}$ [-]	0.095 (0.030)	0.162 (0.207)	0.074 (0.047)	0.075 (0.037) ^∗^
${FD}_{head,ML}$ [-]	1.02 (0.01)	1.02 (0.01)	1.03 (0.02)	1.03 (0.02)
${FD}_{head,AP}$ [-]	1.04 (0.02)	1.04 (0.03)	1.04 (0.03)	1.06 (0.01) ^∗^

* *p* $<0.05$, group effect (Mann-Whitney U-test)

^†^ *p* $<0.05$, effect of the spinning (Wilcoxson’s rank test)

## Discussion

The present paper examined and compared the dynamic balancing ability of Hungarian folk dancers and controls including the effect of spinning activity. It was hypothesized that dancers’ advanced dynamic balancing skills, due to the many years of experience in Hungarian folk dance, would be observable by characterizing the motion of the unstable balance board and the participant’s balance control (CoM and head movements). For this purpose, a comprehensive analysis was carried out involving "classical" (linear) postural stability measures (CoM path, area of the $95\%$ confidence ellipse) and nonlinear measures such as sample entropy and Higuchi’s fractal dimension. Furthermore, the motion of the multiaxial unstable balance board was described by the range AP and ML angles, the percentage of stable ratios, the ratios of AP and ML angles, and the first stable settling time of the two angles.

Regarding the between-subject comparison *before* the spinning intervention, results showed that both groups exhibited similar dynamic balance performance indicated by both the balance board parameters and the linear and nonlinear CoM and head movement parameters. These findings substantiate earlier observations [41, 57, 58], supporting the general idea that the postural control capacities are specific to the training program and demand intrinsic to each discipline. The specialized skills retrieved from the many years of experience in folk dance may not necessarily translate to comparatively less demanding balance conditions as evidenced by the base measurements in the present experiment.

After the spinning intervention, significant intergroup differences emerged. Considering the balance board parameters, the dancing group exhibited enhanced performance in the ML direction (indicated by the roll angle), compared to the control group. This improved ML performance is a key indicator of differing balance capabilities between the two groups, potentially stemming from distinct motor control patterns influenced by biomechanical constraints in the sagittal and frontal planes. Postural control mechanisms vary between the ML and AP directions. The ankle joint predominantly influences AP balance, while body weight is distributed between the heel and the toe, and the board motion is controlled by the activity modulation of flexor/extensor muscles of the lower extremities. On the other hand, when the board oscillates along the ML direction, the body alternatively loads one foot while unload the other. During this loading procedure, the hip’s muscles (abductor/adductor) play a dominant role [59, 60]. Since we did not conduct electromyographic recordings, our data do not provide sufficient support for muscle dynamics-based interpretations. Nevertheless, due to the intrinsic stability of ankle and knee joints in the ML direction, and enhanced hip control through the abduction/adduction synergy, one can anticipate heightened fluctuations along the AP direction among dancers. Considering the nonlinear parameters, it becomes apparent that the significant differences between the two groups primarily manifest in movements along the AP direction (*SampEn* and *FD*). In alignment with the muscle dynamics findings, a reduced *SampEn* value was found in dancers for both head and CoM movements in the AP direction. Previous studies have shown that specialised athlete group (i.e. dancers, gymnasts or ballet dancers) was characterized by more irregular CoP fluctuations (as exemplified by higher *SampEn*), indicating that their balance was somewhat more automatized, implying reduced attention demands compared to their age-matched controls [10, 58, 61]. An essential distinction, however, lies in the fact that these studies focused on static balance tests (and CoP measurements), investigating the influences of visual feedback and varied stances (bipedal and unipedal). Dynamic balancing, on the other hand, entails a more complex challenge; moreover, in our series of measurements, we extended our investigation to encompass the impact of a sport-specific perturbation (spinning intervention). Higuchi’s fractal dimension showed a higher value in the AP direction for both participant groups in comparison to the ML direction. Furthermore, a notable statistical difference in the intergroup variation is observable specifically within the AP direction. The dancing group exhibited increased *FD* values concerning both head and CoM movements. This finding is in line with findings from Casabona et al., who similarly obtained higher *FD* values for the group of ballet dancers during analysis of specified upright postures [62]. Drawing upon previous research, it was deduced that an increased *FD* value can be associated with enhanced adaptation, particularly concerning the sensory-motor system. Consequently, in our case, the dancers’ superior adaptation (stemming from their many years of dance experience) was reflected in the increased *FD* value.

To summarize, the dancers applied a more intricate and resource-intensive strategy (intensive compensation in the ML direction) to address the balancing task, yielding a better balancing performance in terms of balance board parameters. However, taking into account the analysis of the head and CoM movement, it was found that this otherwise expected, more advanced balancing skill of the dancer group is realized somewhat differently as established by prior literature. By preferring solid stability in the ML direction, a greater fluctuation in the AP direction can be observed (indicated by the lower *SampEn* values). However, a comprehensive evaluation of movement complexity, supported by the elevated *FD* values, underscores that the dance group exhibits superior adaptation compared to the control group, even amidst this distinct balancing strategy. Additionally, the inherent nature of Hungarian folk dance accentuates the need for precise and tightly executed movements, even following repeated rotations [2]. Without targeted balance-enhancing training for the dancers, it is probable that a resource-intensive approach involving heightened muscle engagement and attention allocation will be opted for in order to achieve accurate execution.

The overall more successful performance is further evidenced by significant differences observed mostly within the control group in the context of within-subject comparison. An instance that deviates from this pattern is kept in the balance board parameters, where the earlier-noted enhancement in the ML direction is also observable among the dancers. In contrast, for the CoM and head movement descriptors, a notable performance decline is observed within the control group. However, despite the increased path length and area, we can keep an increase in the entropy values after the spinning intervention. This suggests a shift towards a more automated co-origination movement, potentially reflecting an instinct-driven compensation rather than an advanced balance response to intervention. These findings align to some extent with those reported by Livska et al. [14].

An important limitation of the study is that the different dancers are not selected from the same dance ensemble. As a result, the variation in coaching methodologies, training techniques, and exercise frequencies across the groups can potentially impact their balance performance. However, none of the groups underwent specialized balance training as part of their practice in any of the cases. Moreover, Hungarian folk dance ensembles, influenced by various Carpathian Basin dances, exhibit uniqueness in their style and dynamics. This diversity in practice methods poses challenges in forming a cohesive group and quantifying the extent of balance-specific training.

Another limitation of the study is that we must partially rule out the possibility of the control group having undergone balance-specific training. For this group, our sole criterion was that they were not currently engaged in any sport that could impact their balance abilities. Hence, heterogeneities were also observed in this group. Increasing the number of participants can allay such heterogeneities.

In conclusion, our exploration into the effects of dance styles on balance perception contributes to the broader understanding of how dancers consciously develop and apply their balancing skills. By moving beyond the "classical" dynamic balance tests (e.g., Y-Balance Test or Star Exclusion Test) and employing metrics that offer nuanced insights, our research sheds light on the intricate aspects of dancers’ balancing abilities. The integration of rotational fatigue intervention, a common movement in folk dances, allowed us to mimic real-world conditions within a laboratory condition. Future research could benefit from intensified interventions, with participants separated by training levels, to yield a comprehensive evaluation of dancers’ performance and balancing capabilities, thereby informing targeted interventions and training strategies.

The results highlight the significance of targeted balance training as a crucial component in dancers’ preparation, attending heightened awareness of their balance. The measurement methods and evaluations introduced in this study could serve as valuable tools for ongoing tracking and assessment in future studies.

## Conclusions

The study investigated and compared the dynamic balance capabilities of Hungarian folk dancers relative to a control group, considering the effect of spinning intervention. The hypothesis centered on the dancers’ advanced dynamic balancing skills due to their extensive experience in Hungarian folk dance. Initial comparison *before* spinning intervention revealed similar dynamic balance performances between groups in balance board parameters, linear and nonlinear CoM, and head movement parameters. These findings align with previous observations, suggesting discipline-specific postural control capacities. Notably, specialized skills from folk dance may not necessarily translate to less demanding balance conditions. After the spinning intervention, dancers exhibited improved ML performance in balance board parameters, potentially due to distinct motor control patterns influenced by biomechanical constraints. In the AP direction, ankle joint stability and hip control interaction led to anticipated fluctuations among dancers. Significant differences in nonlinear parameters were primarily observed in AP movements, with dancers showing reduced *SampEn* for head and CoM movements. While previous studies focused on static balance tests, this study’s inclusion of spinning intervention demonstrates dynamic balancing complexities. Dancers employed a more resource-intensive strategy (intensive compensation in the ML direction), while controls showed increased entropy values post-intervention, suggesting a shift toward automated co-origination movement. Overall, the results support the dancer group’s superior dynamic balance performance and offer insights into the influence of specific interventions.

## Data Availability

The datasets generated and analyzed during the current study are not publicly available due to the large amount of motion capture data with associated excel files but are available from the corresponding author on reasonable request.

## Abbreviations

AP: Anterior-posterior
CoM: Center of motion
CoP: Center of pressure
FD: Fractal dimension
ML: Medio-lateral
SampEn: Sample entropy
